# Lincolnshire Justices and Belgian Labour Colonies

**Published:** 1904-04-02

**Authors:** 


### Modern Sociology.

### LINCOLNSHIRE JUSTICES AND BELGIAN LABOUR COLONIES.

The justices of the Lindsey Division of Lincolnshire have got tired of punishing vagrants by short sentences to hard labour. A very sufficient reason for this is given in the records of two out of the many tramps who have come before them in the course of their duties. A man, aged 30, has spent nearly three out of the last five years in prison, and the travelling expenses of himself and his escort to and from court-house and gaol have amounted to nearly £12. Another, aged 37, has, since 1886, spent six years in prison, and his travelling expenses amount to £16 10s. This is entirely exclusive of the cost of maintenance of the prisoners. It is therefore clear that short sentences for vagrancy are neither deterrent nor reformatory. Hearing of the methods of treatment in vogue in Belgium for such cases, the justices of Lindsey sent a deputation there to investigate the success of these.

The vagrant is, speaking generally, the inefficient. Some are by birth and inclination predestined recruits for the criminal classes, but the more innocent are simply those who, being less industrious or less skilled than their neighbours, are the most readily dismissed when employment becomes slack, and who, having once tasted the lazy ways of vagabondage are ready to adhere to them or return on slight temptation. As life goes on their inefficiency increases, until at last they are unfit for work even if work were offered them. The Belgian method is meant to stop this downward career at its first stage by placing the tramp at his first appearance in an environment where work will be demanded of him, where he will not lose, but rather increase such skill as he has; where, at the best, he will be fitted to go out again into the world, but where, at the worst, he will lead a harmless and comparatively useful life, exempt from the temptations which hindered his success in independent life. These conditions seem to be met in the penal labour colony as it exists in Belgium.

Of these colonies there are five, two at Bruges for women, and three near Antwerp for men, Hoogstraeten, Wortel, and Merxplas. It was the Merxplas colony to which the Lindsey commissioners devoted most attention, but the conditions in the others are practically the same. The labour colony, however, takes to a great extent the place of the workhouse in England. Thus at Merxplas there are many old men who could do no work, but besides receiving ordinary rations, they are allowed a few centimes a day for the purchase of little luxuries at the canteen of the colony. This seems a very reasonable indulgence for old men, who have led a respectable life, but can no longer keep themselves. The less infirm do light work for which they receive a small payment. These people enjoy a better diet than the ordinary colonist, and can spend their leisure in reading or playing games; but cards are not allowed. Men under 21 who are sent to the colony are kept as much as possible apart from the other colonists, and part of their time is spent in school instead of in the workshops. Another, but very different, special class is that of men sentenced to colony life after imprisonment, or whose past life shows them to be dangerous to the community, and those sentenced for offences against morality. These men are kept apart from the others, and their life resembles that of prisoners, except that they can by their industry earn a small wage.

The typical colonists are those placed in Classes III. and VI. of the division of inmates, viz., "Habitual vagabonds, mendicants, inebriates, and men generally unable to support themselves," and "first offenders." These are expected to do about nine hours work a day, for which they are paid, in addition to their board and lodging, a sum varying from three to thirty centimes a day. This wage is partly handed over at once, in tokens exchangeable only at the canteen at the colony, while part is banked to the credit of the colonist, and is given to him when he leaves. In summer the colonists get up at half-past four in the morning, and after breakfast enter the shops at six. At eight o'clock they have a half-hour's interval to rest and smoke, and then work on till 11.30, when they go in to dinner. They resume work at 1.30, have a half-hour interval at four o'clock, and continue work until six. They then have supper and go to bed. In winter they begin work later, and in the afternoon work by artificial light. "The colonists are given no meat," say the Lindsey investigators, "but the soup of vegetables which we tasted was very good, and each man had a large quantity. They have a sweet drink made of liquorice-wood boiled in water with their meals, coffee and bread for breakfast, potatoes or other vegetables with a meat sauce for supper, and chicory water in large cans in their dormitories. To supplement the above they can make purchases from the canteen of beer, tobacco, lard, herrings, etc., which are sold at exceedingly small prices, representing only the actual cost price of the article when produced by the colony labour itself."

The shops include, besides a farm, an ironfoundry, a tile-making establishment, mat-making, weaving, carpentering, and weaving shops, besides a shoemakers' and a button factory. Only the last competed with the outside world in that the lathes were bought from outside, and the orders came from without the Colony, but on the whole every effort is made to consume within the precincts of State institutions the product of the industry of the colony.

To prevent the risk—which is always present to the mind of the rate-payer—of prison labour competing with that of free men, not only is the work of the colonists sold wherever possible to the State, but as little machinery as possible is used. Where grinding is done a large capstan wheel is used, which takes up the time of, in all, 120 men. The lathes in the shops are worked by driving-wheels turned by men. The looms are all hand-looms. Nothing is done to make work easy in the colony, and the capacity of the unskilled, such as it is, gets every chance it can. As far as possible everything used in the colony itself is made there; not merely clothes, but houses and all else required.

A thing that struck the English observers as strange was that so little objection was made to the almost daily desertions from the place. But it was explained to them that those deserters who had the faculty for self-help would probably obtain occupation outside, while for the rest, their obvious destiny was before long to fall again into the hands of the law, and be sent back to the place from which they had fled. The majority of the colonists in Belgium are the permanently inefficient. But even so, the inhabitant of Merxplas leads a decent life, while the restrictions of the colony prevent that worst of evils, the development of a hereditary inefficient class. The defects of the Merxplas colonists at least die with them. And meanwhile, to quote the report of the investigators, "they have a decent and fairly comfortable life, which is largely self-supporting, and the cost is certainly far less than that of keeping them outside by the agency of charitable doles interspersed with costly periods of residence in workhouse or gaol."

